# Rapid Sputum Multiplex Detection of the *M. tuberculosis* Complex (MTBC) and Resistance Mutations for Eight Antibiotics by Nucleotide MALDI-TOF MS

**DOI:** 10.1038/srep41486

**Published:** 2017-01-30

**Authors:** Kang-Yi Su, Bo-Shiun Yan, Hao-Chieh Chiu, Chong-Jen Yu, So-Yi Chang, Ruwen Jou, Jia-Long Liu, Po-Ren Hsueh, Sung-Liang Yu

**Affiliations:** 1 Department of Clinical Laboratory Sciences and Medical Biotechnology, College of Medicine, National Taiwan University, Taipei, Taiwan; 2 Department of Laboratory Medicine, National Taiwan University Hospital, National Taiwan University College of Medicine, Taipei, Taiwan; 3 Institute of Biochemistry and Molecular Biology, College of Medicine, National Taiwan University, Taipei, Taiwan; 4 Department of Internal Medicine, College of Medicine, National Taiwan University, Taipei, Taiwan; 5 Centers for Disease Control, Taipei, Taiwan; 6 Department of Pathology and Graduate Institute of Pathology, College of Medicine, National Taiwan University, Taipei, Taiwan; 7 Center for Optoelectronic Biomedicine, College of Medicine, National Taiwan University, Taipei, Taiwan; 8 Institute of Medical Device and Imaging, College of Medicine, National Taiwan University, Taipei, Taiwan

## Abstract

The increasing incidence of multidrug-resistant (MDR) and extensively drug-resistant (XDR) *Mycobacterium tuberculosis* (MTB) adds further urgency for rapid and multiplex molecular testing to identify the MTB complex and drug susceptibility directly from sputum for disease control. A nucleotide matrix-assisted-laser-desorption-ionization time-of-flight mass spectrometry (MALDI-TOF MS)-based assay was developed to identify MTB (MTBID panel) and 45 chromosomal mutations for resistance to eight antibiotics (MTBDR panel). We conducted a 300 case trial from outpatients to evaluate this platform. An MTBID panel specifically identified MTB with as few as 10 chromosome DNA copies. The panel was 100% consistent with an acid-fast stain and culture for MTB, nontuberculous mycobacteria, and non-mycobacteria bacteria. The MTBDR panel was validated using 20 known MDR-MTB isolates. In a 64-case double-blind clinical isolates test, the sensitivity and specificity were 83% and 100%, respectively. In a 300-case raw sputum trial, the MTB identification sensitivity in smear-negative cases using MALDI-TOF MS was better than the COBAS assay (61.9% vs. 46.6%). Importantly, the failure rate of MALDI-TOF MS was better than COBAS (11.3% vs. 26.3%). To the best of our knowledge, the test described herein is the only multiplex test that predicts resistance for up to eight antibiotics with both sensitivity and flexibility.

Tuberculosis (TB) is an infectious disease caused by the *Mycobacterium tuberculosis* complex (MTBC) and has always been the top cause of death worldwide^1^. The World Health Organization (WHO) estimated that approximately 9.0 million people developed TB in 2014, and 1.5 million patients died from the disease in 2014^1^. Reports indicate that most new TB cases occur in Asia and Africa and account for 55% and 30% of new cases, respectively. Moreover, the emergence of multi-drug-resistant (MDR) and extensively drug-resistant (XDR) *M. tuberculosis* has added additional threats to public health. Scholars estimate that 3.5% of new cases and 20.5% of recurrent TB cases accounted for approximately half a million cases in 2013 and were caused by MDR-MTB. Among these MDR-MTB cases, approximately 9.7% were XDR-MTB. The capacity of MTB to resist multiple antibiotics has rendered TB treatment difficult and leads to longer hospitalization and higher mortality in patients^2^. Thus, a novel approach for rapidly identifying MTBC and antibiotic susceptibility has become critical for controlling TB.

In clinical laboratories, the rapid identification of MTBC has mainly relied on acid-fast staining, which features a low recovery rate. For example, only 44% of new cases, including 15–20% in children, can be identified by the presence of acid-fast bacilli in sputum smears^3^. Therefore, many commercial or in-house molecular diagnostic methods have been developed to identify MTB. Among these assays, the COBAS TaqMan MTB test (Roche Diagnostics, Branchburg, NJ) is one of the most widely used tests in clinical diagnostics. Another promising diagnostic is GeneXpert MTB/RIF (Cepheid, Sunnyvale, CA), which can simultaneously detect MTB and rifampin resistance directly from sputum within 2 hours^4^. Both assays not only show good sensitivity and specificity but also exhibit certain improvements in the testing procedure, including convenience and turnaround time^4^,^5^,^6^. However, due to the growing incidence of TB cases caused by antibiotic-resistant MTBC, a novel strategy capable of detecting multiple-resistance mutations for different anti-TB drugs directly from raw specimens is necessary.

Previously, we developed a nucleotide matrix-assisted laser desorption ionization time-of-flight mass spectrometry (MALDI-TOF MS) assay consisting of 25-multiplex probes to detect 50 types of four cancer-driving mutations^7^,^8^,^9^. Thus far, this approach has provided routine molecular diagnostics for more than 8000 cases under an ISO15189-certified central laboratory^10^. Here, we extend the MALDI-TOF MS application to tuberculosis diagnoses (see details in Methods) and successfully established a MALDI-TOF-based molecular assay for identifying MTB and resistance mutations for eight anti-TB drugs. In a parallel test using clinical patient sputum specimens, this assay showed better sensitivity and specificity than the COBAS TaqMan MTB test. Thus, the MALDI-TOF-based MTB assay is a promising new strategy for the rapid, simultaneous identification of MTB and drug resistance from sputum.

## Results

### Identifying MTBC and Non-Tuberculosis Mycobacteria (NTM) Using MALDI-TOF MS

**Figure 1:**
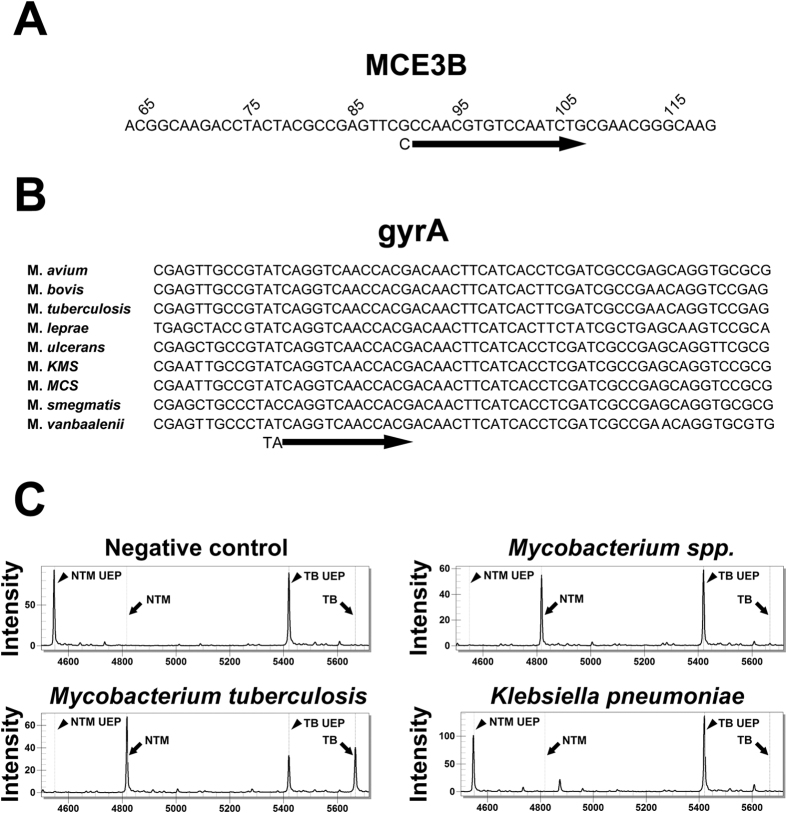
Probe localization for the MALDI-TOF MS MTB and NTM identification and specificity tests. (**A**) Illustration of the MTB-specific detection probe located on the *mce3B* gene locus (Forward). (**B**) Illustration of the common NTM detection probe located on the *gyrA* gene locus (Forward). (**C**) MALDI-TOF MS specificity test. Mass spectra for the negative control, MTB, NTM (Mycobacterium *fortuitum*) and *K. pneumoniae*. A positive signal was identified as a peak shift from UEP to an additional nucleotide mass (represented as TB or NTM). Nucleotides in lower case in (**A**) represented additional mismatch nucleotides for mass adjustment in the following analysis. UEP, unextended probe.

The TB-specific *mce3B* gene or NTM common *gyrA* gene was amplified using PCR specific primers (Supplementary Table 1) followed by a single nucleotide extension reaction using “TB”- or “NTM”-specific probes (Fig. 1A,B and Supplementary Table 2). Probes without a single nucleotide incorporated are referred to as unextended probes (UEPs) and did not produce a mass-shifted signal. Due to the significant differences in the probe masses, the two probes can be detected in a single reaction (Reaction #1 in Supplementary Table 2) (Fig. 1C). To evaluate the specificity of the platform, genomic DNA from several reference strains, including *M. tuberculosis* H37Ra, 7 strains of *Mycobacterium* spp. and 5 non-Mycobacterium bacterial stains, were assayed in the pilot study (Supplementary Table 3). As shown, only the *M. tuberculosis* genomic DNA spectrum showed shifted signals for both the TB and NTM probes (Fig. 1C, lower left panel). In contrast, the *Mycobacterium fortuitum* and other NTM genomic DNA spectra exhibited shifted signals for the NTM probe only (Fig. 1C, upper right panel and Supplementary Fig. 1A). Moreover, the *K. pneumoniae* and other non-mycobacterium bacterial genomic DNA spectrum did not exhibit shifted signals for either the TB probe or the NTM probe, which indicates that the assay is highly specific for mycobacteria (Fig. 1C, lower right panel and Supplementary Fig. 1B). Taken together, MALDI-TOF MS can specifically distinguish *M. tuberculosis*, non-tuberculosis mycobacteria and non-mycobacterium bacteria in multiplex reactions.

### Limit of Detection Test for MALDI-TOF MS

**Figure 2:**
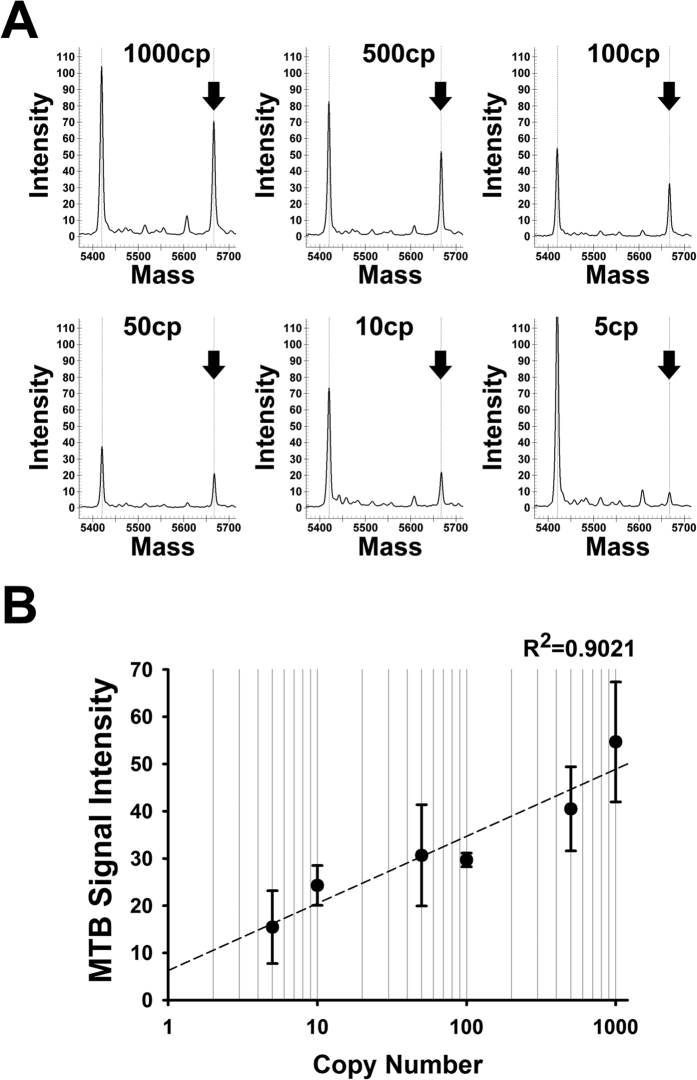
MALDI-TOF MS detection limit for MTB identification. Genomic DNA extracted from *M. tuberculosis* H37Ra was serially diluted from 1000 to 5 copies in distilled water. The signal height was obtained using Type4 software. (**A**) Mass spectra of serial dilutions with various MTB copies. Positive signals are indicated by arrows. (**B**) Correlation between the diluted MTB copy number (theoretical copy number) and positive signal height from MALDI-TOF MS.

According to the results of serially diluted MTB genomic DNA testing, as shown in Fig. 2A, the TB probe’s shifted signal can be detected with as low as 5 MTB genomic DNA copies. The signal intensity correlates highly with the MTB genomic DNA copies, as demonstrated by the linear correlation value (R squared) at 0.902 (Fig. 2B).

### Comparing Results from Acid-fast Staining, Bacterial Culture, and MALDI-TOF MS

We further performed a double-blind test with clinical isolates cultured from sputum specimens using an MTBID panel, acid-fast staining and traditional bacterial culture to identify MTB and NTM (Table 1). The results indicate that MALDI-TOF MS can detect the culture-positive MTBC cases of both TB and NTM with 100% consistency. However, acid-fast staining failed to identify 3 of 5 and 3 of 6 culture-confirmed TB and NTM cases, respectively.

### Detecting Drug Resistant Mutations Using MALDI-TOF MS

In addition to MTB identification, we also designed an MTBDR panel with specific probes for detecting mutations in several resistance-associated genes, including *rpoB* (codon 513, 516, 522, 526, 531 and 533), *katG* (codon 315), and *inhA* (promoter -15 nucleotide) (Table 2). To evaluate the feasibility and accuracy of the MTBDR panel, twenty MDR-MTB strains with known antibiotic-resistance mutations were obtained from the Taiwan CDC and assessed using the MALDI-TOF MS platform and Sanger sequencing in a double-blind study. As shown, all mutations detected using the MALDI-TOF MS assay were also identified through Sanger sequencing. The results were 100% consistent.

**Figure 3:**
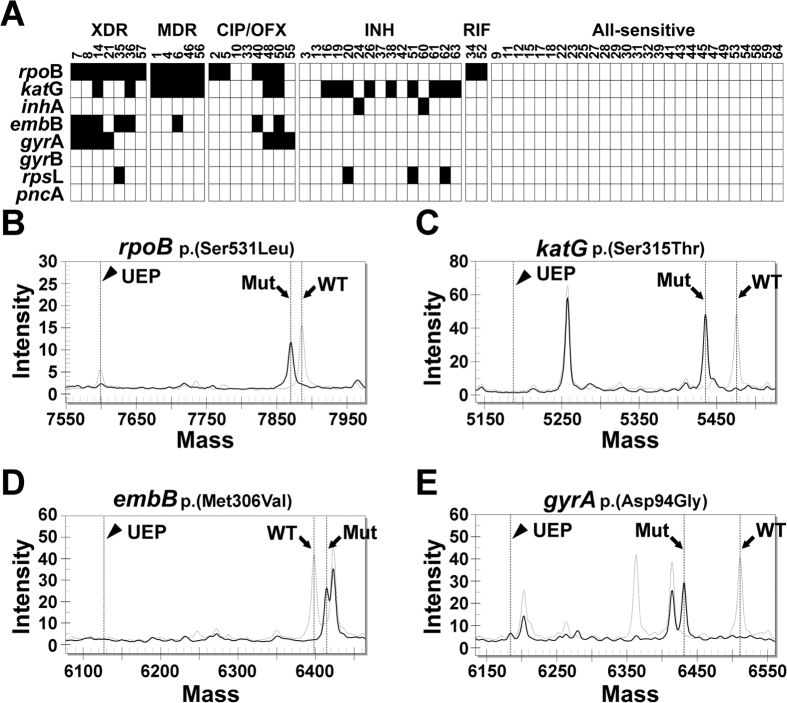
Correlation of drug-resistant gene mutations measured using the MALDI-TOF MS and culture-based drug susceptibility tests. (**A**) Sixty-four clinical isolates with drug susceptibility test results were assessed for the drug-resistant gene mutation test using MALDI-TOF MS. Mutations were not identified in the all-sensitive drug isolates. Six isolates resistant to drugs did not exhibit a mutation using our testing panel. Mutations in the *rpoB* gene included five isolates at codon 516, one at codon 526, and 13 at codon 531; mutations in the *katG* gene were all at codon 315. Mutations in the *inhA* gene were all at the −15 nucleotide position, and mutations in the *embB* gene were all at codon 306. Mutations in the gene *gyrA* included two at codon 90 and five at codon 94, and mutations in the *rpsL* gene included one isolate at codon 88 and three at codon 43. (**B–E**) The mass spectrum of XDR-MTB (#14) (black solid line) overlapped with all-sensitive MTB (#9) (grey dotted line) and exhibited mutations in four genes, including *rpoB* (Ser531Leu) (**B**), *katG* (Ser315Thr) (**C**), *embB* (Met306Val) (**D**), and *gyrA* (Asp94Gly) (**E**).

Furthermore, 64 clinical MTB isolates with known antibiotic susceptibility, including seven XDR-MTB, five MDR-MTB, 8 fluoroquinolone-resistant MTB, 15 isoniazid-resistant MTB, 2 rifampin-resistant MTB and 27 all-sensitive MTB, were obtained from the NTUH for the MTBDR panel validation (Fig. 3A). Among 45 probes for 8 first- and second-line anti-MTB drugs, no mutations were detected among the 27 all-sensitive isolates. MALDI-TOF MS had 90.6% concordance with the drug susceptibility test. Four isoniazid-resistant isolates (#3, #13, #37, and #42) and 2 ofloxacin-resistant isolates (#10 and #33) did not test positive for any of the selected probes. Moreover, the XDR-MTB clinical isolate (#14) mutation spectra were clearly distinguished from the all-sensitive spectra in the *rpoB, katG, embB*, and *gyrA* genes (Fig. 3B–E). Using the drug-susceptibility test as the reference method, the sensitivity and specificity of our MTBDR panel are 83.8% (31/37) and 100% (27/27), respectively.

### MALDI-TOF MS Performance Directly Using Sputum Specimens

Among the 300 samples tested, 79 samples were undetermined/invalid by COBAS (26.3%) testing, while only 34 samples were not identified by MALDI-TOF MS (11.3%) testing. Using bacterial culture as the reference method for identifying MTB, the COBAS vs. MALDI-TOF MS sensitivity and specificity were 46.6% vs. 61.9% and 96.0% vs. 92.4%, respectively, for the smear acid-fast bacillus-negative cases (Table 3). Concerning the 11 smear AFB-positive cases, the MALDI-TOF MS vs. COBAS sensitivities and specificities were comparable. In addition to MTB identification, MTBID panel-positive samples from the MALDI-TOF MS assay were further assessed using the MTBDR panel. No drug-resistance mutations were detected among these samples, which was consistent with the drug susceptibility test. Overall, the COBAS vs. MALDI-TOF MS sensitivities and specificities were 52.6% vs 72.4% and 96.0% vs. 92.4%, respectively (Table 3).

## Discussion

The nucleotide MALDI-TOF MS test is a simple, rapid and multiplexed method with high specificity, sensitivity and flexibility in many research fields, including mutation and SNP analysis, sequencing, and microorganism detection^11^,^12^,^13^,^14^,^15^,^16^,^17^,^18^. Compared with other detection systems, MALDI-TOF MS acquires the absolute mass value, which represents an intrinsic property of a molecule, while others depend on signals of a relative electrophoretic mobility or a hybridization event. In addition, the molecular weight is a significantly more informative signal than fluorescent label-based methods. This study applied this platform to establish a rapid multiplexed test to detect MTB and its drug susceptibility in sputum. When testing for MTB at an early stage, the sensitivity, specificity, positive prediction value (PPV), and negative prediction value (NPV) are comparable with other currently used methods (Table 3). Compared with the limit of traditional testing methods such as the sensitivity of microscopic examination (22–65%) or bacillus culture (approximately 10–20% unsuccessful), MALDI-TOF MS provided a solution for disease control and management^15^,^19^,^20^. Furthermore, the rapid diagnosis of MTB infections is critical for successful treatment. However, due to the limit of detection, the low sensitivity impedes rapid laboratory MTB diagnosis in patients. Many PCR-based molecular diagnostic methods have been developed in recent years. However, the sensitivity of the assays remains unsatisfactory, particularly for acid-fast stain negative samples in which only 43–74% of samples were identified^21^,^22^. Our results show that the MALDI-TOF MS detection limit is less than 10 MTB copies (Fig. 2). This characteristic facilitates MTB detection in raw specimens, including sputum, and significantly reduces the turnaround time for diagnosis.

Another advantage of MALDI-TOF MS is the panel flexibility for drug-resistant MTB prediction based on customized requirements, which are widely considered serious threats to global TB control. In this panel, we simultaneously predicted resistance for anti-TB drugs, including, rifampin, isoniazid, pyrazinamide, streptomycin, ethambutol, fluoroquinolone, pyrazinamide and ethionamide. This test can identify drug-resistant MTB more comprehensively than the rifampin-resistant MTB determined using GeneXpert MTB/RIF. Among 64 clinical isolates, all sensitive or drug-resistant strains could be identified using our system (Fig. 3), which could provide a rapid diagnosis compared with the traditional six- to eight-week bacterial culture process. Because there was no mutation detected in six resistant isolates (Fig. 3, #10, #33, #3, #13, #37, and #42), it was possible that other rare mutations may not be included in our panel, and any other unknown resistance-mechanisms may not be detected^23^,^24^,^25^. The limitation of this platform is that MALDI-TOF MS cannot perform de novo mutation identification; however, adding novel drug resistant mutations into the multiplex panel is a convenient process. Any newly identified mutations can be detected by designing a specific probe and coordinating with other probes in the spectrum. Thus, MALDI-TOF MS has a greater capacity compared with other molecular diagnostic methods, such as the INNO-LiPA Rif TB kit (LiPA) (Innogenetics, Zwijndrecht, Belgium)^26^. Furthermore, due to the single nucleotide extension reaction in the biochemical process, this assay can detect various alterations within one nucleotide residue that other molecular methods cannot detect, such as ARMS or COBAS^27^.

The other greatest challenge for MTB identification is early diagnosis and short turnaround time. Regarding the time-consuming culture process, many well-established sensitive systems have been developed to improve detection rates at early stages, such as COBAS and GeneXpert MTB/RIF. With the development of new methods, sputum specimens have been routinely used in clinical diagnoses. One important issue should be considered that may cause false negative results using sputum for MTB identification: the copy number of MTB in the sputum, especially in smear-negative sputum. The overall sensitivity and specificity of COBAS ranged from 66.9% to 82.4% and 97.7% to 100%, respectively^5^,^6^,^28^. However, the sensitivity for smear-negative, culture-positive samples was relatively low (34.4–73.6%). For the smear-negative cases in this study, MALDI-TOF MS showed 61.9% sensitivity and 92.4% specificity, which is better than COBAS and comparable to other systems^5^,^29^,^30^. Methodologically, despite the concern of multiplex detection, TaqMan QPCR-based COBAS required relatively high quality nucleic acids, not only in large amounts but also of good integrity, for probe annealing. That means samples with highly degraded nucleic acid or reaction inhibitors may result in a poor reaction and an undetermined/invalid result. In addition, the unpredicted nucleotide alteration within the probe binding region may affect detection. This may be the cause of the failure rate of MALDI-TOF MS and COBAS in this study (11.3% vs 26.3%) (Table 3). The sensitivity of GeneXpert MTB/RIF ranged from 43.4% to 76.9% in smear-negative, culture-positive specimens^4^,^5^,^29^,^31^,^32^,^33^,^34^. MALDI-TOF MS was also competitive with this method. However, for clinical utility, using GeneXpert MTB/RIF might be restricted by the limited shelf-life of the cartridges, strict environmental considerations or high-level hardware requirements^35^,^36^. Thus, MALDI-TOF MS may provide a more friendly platform for TB management than other methodologies.

Finally, the PPV and the NPV are also important for clinical practice. In this study, the PPV of the pooled specimens was unsatisfactory (Table 3) due to a low prevalence rate in the unselected patient population. It should be noted that only 3.6% of samples were smear-positive in this study. Although this is similar to another study that examined a large number of specimens, we think a more comprehensive prospective study is necessary for further clinical validation^37^. MALDI-TOF MS may be suitable for low-prevalence developing countries. Taken together, we developed a MALDI-TOF MS-based highly sensitive molecular testing method that combines MTB identification and drug-resistance predictions. It should be emphasized that the flexibility will allow clinicians to rearrange or modify the detection panels based on clinical needs. Additionally, to the best of our knowledge, this is the only platform that comprehensively features multiplex detection for most MTB drug-resistant mutations, not only for rifampin resistance. Further, the turnaround time was less than 48 hours from sample receipt to data analysis, which is also comparable to other methods^38^. This method provides a powerful and an alternative strategy for MTB disease management and control.

## Methods

### Study Cases

This study was conducted at the National Taiwan University Hospital (NTUH), which is a 2500-bed medical centre in Taipei, Taiwan. A total of 300 consecutive and non-duplicate sputum samples that were submitted to the Mycobacteriology Laboratory of NTUH for mycobacterial isolation from Dec-2010 to July-2011 were evaluated. All experimental procedures with the patients’ specimens and information were approved by the institutional review board of the National Taiwan University Hospital, Taipei, Taiwan (Approval No. 201009064 R, 201103027RC), and all methods were performed in accordance with the relevant guidelines and regulations. All patients with suspected pulmonary TB were enrolled with written informed consent from March 2007 to December 2009. Medical records were reviewed, and data were collected on age, sex, underlying diseases, pathology and microbiologic results, and follow-up observations. A subject was categorized as a confirmed TB patient only if MTBC was recovered by culture. Patients with TB-related clinical symptoms and signs only or clinical improvement without anti-TB therapy were considered non-confirmed TB cases.

### Reference Strains and Clinical Isolates of MTBC

Bacterial strains, including *M. tuberculosis* (H37Rv), *Mycobacterium* spp. and non-mycobacterium bacteria, were obtained from the Bioresource Collection and Research Center (BCRC) (Hsinchu, Taiwan) (Supplementary Table 3). All clinical isolates were acquired from the NTUH and the Center of Disease Control (CDC), Taiwan.

### Laboratory Examination, Sputum DNA Extraction and TaqMan MTB

The laboratory examination and sputum DNA extraction methods are detailed in the Supplemental Data. TaqMan MTB was performed using COBAS^®^ TaqMan 48 (Roche Diagnostics) according to the manufacturer’s instructions. Briefly, 50 μl of the extracted DNA solution was mixed with 50 μl of the amplifier mix containing TaqMan MYCO magnesium reagent, TaqMan MYCO internal control, and TaqMan MTB master mix for TaqMan QPCR. The quantity of MTB can be determined by calculating the difference between the Ct value of the positive control (Ct/p) and that of the sample (Ct/s) by using the formula: MTB = 2^(Ct/p − Ct/s) × 20. The resulting interpretation was according to the instructions for *in vitro* diagnosis (Doc Rev. 4.0). All raw data calculations utilized AMPLILINK software Version 3.3 (Roche Diagnostics). The results of individual specimens that passed the criteria of the negative control, positive control, and internal control were defined as a valid run. MTB was determined to be detected or not detected only in a valid, but not invalid, run.

### Mutation Detection by MALDI-TOF MS

All nucleotide mass spectrometry assays utilized the MassARRAY^®^ System (Cat. No. 10411, SEQUENIM, San Diego, CA acquired by Agena Bioscience, http://agenabio.com/, San Diego, CA at 2014). It combined mass spectrometry and sensitive and robust chemistry in genetic testing. Briefly, for mutation detection, the desired region of interest was PCR-amplified using specific primers followed by shrimp alkaline phosphatase (SAP) treatment to remove excess dNTP. An iPLEX^®^ Pro (Cat. 10217, Agena Bioscience, San Diego, CA) single nucleotide extension was performed using the home-designed probe to distinguish the mutant nucleotide from the wild-type due to the mass difference. The iPLEX^®^ biochemical reaction products were dispensed onto the SpectroCHIP^®^ Array using the MassARRAY^®^ Nanodispenser RS1000, a silicon chip with a pre-dispensed matrix crystal. Finally, the chip was placed into the MassARRAY^®^ Analyzer 4 MALDI-TOF MS and analysed.

### Biochemical Reaction for MALDI-TOF MS Analysis

TB identification and antibiotic-resistance mutation detection were performed using MassARRAY^®^ System and iPLEX^®^ Pro following the manufacturer’s protocol. Briefly, a total volume of 5 μl of a mixture containing 10 ng of bacteria or sputum DNA, 0.5 unit HotStarTaq DNA polymerase, 500 μM dNTPs, 100 nM of specific primers (Supplementary Table S1), 1.25 μl of 10× HotStar buffer and an additional 1.625 mM MgCl_2_ was subjected to PCR reactions with conditions as follows. A single activation cycle at 94 °C for 15 min was followed by 45 touch-down amplification cycles, consisting of 15 cycles of 94 °C for 20 sec, 61 °C annealing for 30 sec, 72 °C for 60 sec and another 30 cycles with 57 °C annealing for 30 sec. The PCR products were then treated with SAP for dNTP neutralization as follows: 0.5 units of SAP with 1.7× SAP buffer were prepared into a final volume of 2 μl of mix and then added to the PCR product for 40 min of incubation at 37 °C, followed by 5 min of inactivation at 80 °C. Next, the SAP-treated PCR products were subjected to the single nucleotide extension reaction by using an iPLEX Pro^®^ reagent kit containing 0.04 μl of Sequenase, 0.1 μl of termination mix, 0.2 μl of 10× iPLEX Pro^®^ buffer and multiplex extension primers specific to MTBC, NTM and resistance mutation sites (Supplementary Table 2) with a final concentration of 7 to 14 μM in a total of 2 μl of the mixture. Temperature cycling consisted of a modified 60 °C annealing and 200-cycle extension method (94 °C, 30 sec followed by 40 repeats of 5 rounds of 94 °C for 20 sec, 80 °C for 5 sec, 60 °C for 5 sec). After desalting with SpectroClean Resin, the samples were loaded onto the matrix of the SpectroCHIP^®^ using a MassARRAY^®^ Nanodispenser RS1000 and then analysed using a Bruker Autoflex MassARRAY^®^ Analyzer 4 MALDI-TOF MS. Data were collected and analysed using Type4 software (SEQUENOM). The signal in the correct mass position (corresponding to products), which passed the criteria of the signal to noise ratio with an acceptable probability, was interpreted as a positive result by the Type4 software. To confirm that the testing run was valid in sputum, the probe for the human epidermal growth factor receptor (EGFR) gene previously used was spiked into each test as an internal reaction control^8^,^9^. The “undetermined” result representing MTB detection was uncertain due to the poor reaction or other confounding factors.

### Evaluation of Detection Sensitivity for MTB by MALDI-TOF MS

To evaluate the MALDI-TOF MS detection sensitivity, *M. tuberculosis* (H37Ra) genomic DNA was serially diluted in water and used for the assessment. The MALDI-TOF MS detection limit was defined as the highest dilution with signal peaks that could be identified using Type4 software.

### Experimental Design

To optimize the platform, standard bacterial strains including MTB, NTM, and non-mycobacterium bacteria were used for specificity testing, and serially diluted *M. tuberculosis* H37Ra genomic DNA was used for limit of detection (LOD) determination.

The molecular testing was composed of two steps: MTB identification (MTBID panel) (Reaction #1 in Supplementary Table 2) and drug-resistant mutation detection (MTBDR panel) (Reaction #1 and #2 in Supplementary Table 2). Only MTBID-positive samples were further assessed for resistance mutations. Clinical isolates, including drug sensitive MTB, drug resistant MTB (MDR and XDR), and NTM, were used to validate MTBID and the MTBDR panel, while bacterial culture, a drug susceptibility test, and Sanger sequencing were used as reference methods for double blind comparison. For clinical practice, a trial of 300 consecutive and non-duplicate sputum samples were selected to evaluate the MALDI-TOF MS approach in comparison with the COBAS TaqMan method. The traditional laboratory examination was used as the reference.

## Additional Information

**How to cite this article:** Su, K.-Y. *et al*. Rapid Sputum Multiplex Detection of the *M. tuberculosis* Complex (MTBC) and Resistance Mutations for Eight Antibiotics by Nucleotide MALDI-TOF MS. *Sci. Rep.* **7**, 41486; doi: 10.1038/srep41486 (2017).

**Publisher's note:** Springer Nature remains neutral with regard to jurisdictional claims in published maps and institutional affiliations.

## Supplementary Material

Supplementary Information

## Figures and Tables

**Table 1:** Comparison of Mycobacterial Identification using Acid-Fast Staining, Bacterial Culture and MALDI-TOF MS.

patient ID	acid-fast bacilli	culture examination	rifampicin susceptibility	MALDI-TOF MS		
				TB	NTM	*rpoB* mutation
2986	+	*M. tuberculosis*	sensitive	+	+	−
3012	+	*M. tuberculosis*	sensitive	+	+	−
2994	−	*M. tuberculosis*	sensitive	+	+	−
2996	−	*M. tuberculosis*	sensitive	+	+	−
3014	−	*M. tuberculosis*	sensitive	+	+	−
2673	+	*M. kansasii*	NA	−	+	NA
2852	+	MAC	NA	−	+	NA
2834	+	*M. chelonae*	NA	−	+	NA
3013	−	*M. kansasii*	NA	−	+	NA
2993	−	*M. gordonii*	NA	−	+	NA
3009	−	MAC	NA	−	+	NA
8288	−	negative	NA	−	−	NA
8289	−	negative	NA	−	−	NA
8290	−	negative	NA	−	−	NA

MAC, Mycobacterium avium complex.

**Table 2:** Comparison of Drug Resistant Gene Mutation Detection in Clinical Isolates by Sanger Sequencing and MALDI-TOF MS.

Sample ID	MALDI-TOF MS								Drug Susceptibility Test			Sanger Sequencing	
	*rpoB*	*katG*	*embB*	*gyrA*	*gyrB*	*rpsL*	*inhA*	*pncA*	Result	RIF	INH	Mutation for RIF	Mutation for INH
MTB-01	533	315							MDR	R	R	*rpoB* codon 533 CTG/CCG	*katG* codon 315 AGC/AAC
MTB-02	531		497			43	−15		MDR	R	R	*rpoB* codon 531 TCG/TTG	*inhAr -*15 C/T
MTB-03	531	315				43			MDR	R	R	*rpoB* codon 531 TCG/TTG	*katG* codon 315 AGC/AAC
MTB-04	522		306				−15		MDR	R	R	*rpoB* codon 522 TCG/TTG	*inhAr -*15 C/T
MTB-05	526								MDR	R	R	*rpoB* codon 526 CAC/TAC	
MTB-06	526	315							MDR	R	R	*rpoB* codon 526 CAC/TAC	*Kat G* codon 315 AGC/ACC
MTB-07	513		406			43			MDR	R	R	*rpoB* codon 513 Q513P/L	
MTB-08	531	315					−15		MDR	R	R	*rpoB* codon S531L	*inhAr -*15 C/T
MTB-09	526	315							MDR	R	R	*rpoB* codon H526D	*katG* codon 315 AGC/ACC
MTB-10	526	315							MDR	R	R	*rpoB* codon H526D	*katG* codon 315 AGC/ACC
MTB-11	531		306						MDR	R	R	*rpoB* codon S531L	INH: WT
MTB-12									N	S	S	*rpoB*: WT	INH: WT
MTB-13		315							MDR	R	R	*rpoB*: WT	*katG* codon 315 AGC/ACC
MTB-14		315							N	S	R	*rpoB*: WT	*katG* codon 315 AGC/ACC
MTB-15		315							N	S	R	*rpoB*: WT	*katG* codon 315 AGC/ACC
MTB-16	516		497						MDR	R	R	*rpoB* codon D516V	
MTB-17	531	315	306				−15		MDR	R	R	*rpoB* codon S531L	*inhAr -*15 C/T
MTB-18	531								MDR	R	R	*rpoB* codon S531L	
MTB-19	531	315							MDR	R	R	*rpoB* codon S531L	*katG* codon 315 AGC/ACC
MTB-20									N	S	S	*rpoB*: WT	ND

RIF, Rifampin; INH, Isoniazid.

**Table 3:** Comparison of MTB identification by MALDI-TOF MS and Roche COBAS QPCR (n = 300).

Detection Method	MTB Results	All Specimens (n = 300)							Smear-Negative Specimen (n = 289)							Smear-Positive Specimen (n = 11)						
		MTB Culture Results							MTB Culture Results							MTB Culture Results						
		+	−	Total	Sensitivity (%)**	Specificity (%)***	PPV (%)	NPV (%)	+	−	Total	Sensitivity (%)**	Specificity (%)***	PPV (%)	NPV (%)	+	−	Total	Sensitivity (%)**	Specificity (%)***	PPV (%)	NPV (%)
COBAS Taqman																						
	+	10	8	18					6	8	14					4	0	4				
	−	22 (13)*	260 (66)*	282 (79)*					16 (9)*	259 (65)*	275 (74)*					6 (4)*	1 (1)*	7 (5)*				
	Total	32	268	300	52.6	96.0	55.6	95.6	22	267	300	46.6	96.0	42.9	96.5	10	1	300	66.7	100.0	100.0	50.0
MALDI-TOF MS																						
	+	21	18	39					13	18	31					8	0	8				
	−	11 (3)*	250 (31)*	261 (34)*					9 (1)*	249 (31)*	258 (32)*					2 (0)*	1 (0)*	3 (0)*				
	Total	32	268	300	72.4	92.4	53.8	96.5	22	267	300	61.9	92.4	41.9	96.5	10	1	300	80.0	100.0	100.0	33.3

MTB, Mycobacteria tuberculosis; QPCR, Quantitative polymerase chain reaction; PPV, Positive prediction value; NPV, Negative prediction value.

^*^The number in the bracket represented “undetermined/invalid” results.

^**^The sensitivity was calculated as “(No. of positive by detection method)/(total No. of MTB culture positive after undetermined/invalid No. in the bracket subtracted) × 100%”.

^***^The specificity was calculated as “(No. of negative by detection method after undetermined/invalid No. in the bracket subtracted)/(total No. of MTB culture negative after undetermined/invalid No. in the bracket subtracted) × 100%”.
