# Supplementary material for: Rapid Sputum Multiplex Detection of the M. tuberculosis Complex (MTBC) and Resistance Mutations for Eight Antibiotics by Nucleotide MALDI-TOF MS
Source: Sci Rep. 2017 Jan 30;7:41486. doi: 10.1038/srep41486 (PMC5278408; doi:10.1038/srep41486)
Supplement: Supplementary Information [file srep41486-s1.doc]

**Supplementary information**

**Rapid Sputum Multiplex Detection of the *M. tuberculosis* Complex (MTBC) and Resistance Mutations for Eight Antibiotics by Nucleotide MALDI-TOF MS**

Kang-Yi Su, Bo-Shiun Yan, Hao-Chieh Chiu, Chong-Jen Yu, So-Yi Chang, Ruwen Jou, Jia-Long Liu, Po-Ren Hsueh^*^, Sung-Liang Yu^*^

*These authors contributed equally.

**Supplementary Methods**

**Laboratory Examination**

For acid-fast bacilli testing, sputum smears were stained with a fluorochrome dye as well as auramine-rhodamine and examined using standard procedures. The fluorochrome-positive smears were further validated using a Kinyoun-staining method. To recover MTB via culturing, 0.5 ml of sediment from a processed sputum specimen was inoculated onto Middle brook 7H11 selective agar (Remel, Lexena, KS)^1^ or into mycobacteria culture tubes in a BACTEC MGIT 960 system (Becton-Dickinson Diagnostic Instrument Systems, Sparks, MD). We identified *M. tuberculosis* and non-tuberculous mycobacteria (NTM) species from the culture using routine, conventional biochemical identification methods.

**Sputum DNA Extraction**

The MTB’s genomic DNAs were extracted from sputum using High Pure PCR Template Preparation Kit (Roche Diagnostics, Branchburg, NJ) in accordance to manufacturer’s protocol. Briefly, after centrifugation for 10 min at 14,000 g, the supernatant of decontaminated sputum was discard followed by addition of 200 μl binding buffer and 40 μl proteinase K solution. After incubation at 70°C for 2 h, the sample was treated with six freeze-and-thaw cycle (4 min in boiling water and 2 min in liquid nitrogen), and genomic DNAs were collected by isopropanol precipitation.

**Supplementary Figure Legend**

**Supplementary Fig. 1.** Specificity test of MALDI-TOF MS in MTB, NTM, and non-mycobacterium bacteria. (A) The spectra for the 7 NTM strains. A positive signal was identified as a peak shift from NTM UEP to an additional nucleotide mass (represented as NTM). (B) the spectra for the 5 non-mycobacterium strains. No positive signal was identified neither TB nor NTM probe. UEP, unextended probe

**Supplementary table**s

| **Supplemental Table 1:** Primer sequence of PCR reaction for MAIDL-TOF MS detection | | | |  |
| --- | --- | --- | --- | --- |
| Gene | Mutation related to drug resistance | primer ID | Sequence* (5’🡪3’) | |
|  | Codon |  |  |  |
| *MCE3B* | n.a | TB_PCRF | acgttggatgTTCCTGCTGATTGCCGTCTTC | |
|  |  | TB_PCRR | acgttggatgTGTCGTAGCGGATCACCGC | |
| *gyrA* | n.a | NTM_PCRF | acgttggatgARYTGCCSTATCAGGTCAAC | |
|  |  | NTM_PCRR | acgttggatgCGSACCTGYTCGGCGATCG | |
| *katG* | 315, 316 | katG_PCRF | acgttggatgTGGAAGAGCTCGTATGGCAC | |
|  |  | katG_PCRR | acgttggatgCTGTTGTCCCATTTCGTCGG | |
| *pncA* | 57 | pncA_PCRF | acgttggatgCAACCAAGGACTTCCACATC | |
|  |  | pncA_PCRR | acgttggatgACGAGGAATAGTCCGGTGTG | |
| *embB* | 306 | embB306_PCRF | acgttggatgAATTCGTCGGACGACGGCTA | |
|  |  | embB306_PCRR | acgttggatgACCAGCGGAAATAGTTGGAC | |
| *embB* | 406 | embB406_PCRF | acgttggatgCGTGGATGCCGTTCAACAAC | |
|  |  | embB406_PCRR | acgttggatgTCGATCAGCACATAGGTGAC | |
| *embB* | 497 | embB497_PCRF | acgttggatgTCATCCTGACCGTGGTGTTC | |
|  |  | embB497_PCRR | acgttggatgGATTTTGGCGCGAACCCTG | |
| *rpsL* | 43 | rpsL43_PCRF | acgttggatgTATGCACCCGCGTGTACACC | |
|  |  | rpsL43_PCRR | acgttggatgACCTGACTCGTCAACTTCAC | |
| *rpsL* | 88 | rpsL88_PCRF | acgttggatgAACCTGCAGGAGCACTCGAT | |
|  |  | rpsL88_PCRR | acgttggatgGATGATCTTGTAGCGCACAC | |
| *rpoB* | 513, 516, 522, 526, 531, 533 | rpoB_PCRF | acgttggatgGATCAAGGAGTTCTTCGGCA | |
|  |  | rpoB_PCRR | acgttggatgACGCTCACGTGACAGACCG | |
| *gyrA* | 90 | gyrA90_PCRF | acgttggatgAGACCATGGGCAACTACCAC | |
|  |  | gyrA90_PCRR | acgttggatgTCCACCAGCGGGTAGCGCA | |
| *gyrA* | 94 | gyrA94_PCRF | acgttggatgAGACCATGGGCAACTACCAC | |
|  |  | gyrA94_PCRR | acgttggatgTCCACCAGCGGGTAGCGCA | |
| *gyrB* | 180 | gyrB_PCRF | acgttggatgGCGGCAAGATCATCAATGTG | |
|  |  | gyrB_PCRR | acgttggatgCGTGATGATCGCCTGAACTT | |
| *inhA* | nucleotide -15 | inhA_PCRF | acgttggatgTAACCAGAATCCGTTTGCCG | |
|  |  | inhA_PCRR | acgttggatgTCGACGGCGGCATGGGTAT | |

*, The nucleotide sequence “acgttggatg” in lower case represents the tag of PCR primers for mass adding. This avoids to interfere the probe interpretation within the mass spectrum.

|  | | **Supplemental Table 2.** Multiplex Probe Panel for Mycobacteria *tuberculosis* Identification and Drug Resistance Prediction | | | | | | | | | |
| --- | --- | --- | --- | --- | --- | --- | --- | --- | --- | --- | --- |
| Reaction | Anti-TB drug | | Gene | Mutation related to drug resistance | | probe ID | Probes/Products of single nucleotide extension sequence* (5’🡪3’) | | Interpretation | Mass (Da) |  |
|  |  |  |  | Codon | Nucleotide alteration |  |  |  |  |  |  |
| 1 | n.a | | *mce3B* | n.a | n.a | TB | cCCAACGTGTCCAATCTG | F | UEP | 5419.6 |  |
|  |  |  |  |  |  |  | cCCAACGTGTCCAATCTG**C** | F | MTB | 5666.7 |  |
| 1 | n.a | | *gyrA* | n.a | n.a | NTM | taCAGGTCAACCACG | F | UEP | 4548.9 |  |
|  |  |  |  |  |  |  | taCAGGTCAACCACG**A** | F | NTM | 4817.2 |  |
| 2 | Isoniazid  (INH) | | *katG* | 315 | AGC-->GGC | katG315_1_AG | gAAGGACGCGATCACC | F | UEP | 4900.2 |  |
|  |  |  |  |  |  |  | gAAGGACGCGATCACC**A** | F | WT | 5171.4 |  |
|  |  |  |  |  |  |  | gAAGGACGCGATCACC**G** | F | Mut | 5187.4 |  |
| 2 |  | | *katG* | 315 | AGC-->AGG | katG315_3_CG | GGACGCGATCACCAG | F | UEP | 4587.0 |  |
|  |  |  |  |  |  |  | GGACGCGATCACCAG**C** | F | WT | 4834.2 |  |
|  |  |  |  |  |  |  | GGACGCGATCACCAG**G** | F | Mut | 4874.2 |  |
| 2 | Pyrazinamide (PZA) | | *pncA* | 57 | CAC-->GAC | pncA169_CG | GTCCGGTGTGCCGGAGAAGT | R | UEP | 6214.0 |  |
|  |  |  |  |  |  |  | GTCCGGTGTGCCGGAGAAGT**G** | R | WT | 6501.2 |  |
|  |  |  |  |  |  |  | GTCCGGTGTGCCGGAGAAGT**C** | R | Mut | 6461.2 |  |
| 2 | Ethambutol (EMB) | | *embB* | 306 | ATG-->GTG | embB306_1_AGC | aaGACGGCTACATCCTGGGC | F | UEP | 6127.0 |  |
|  |  |  |  |  |  |  | aaGACGGCTACATCCTGGGC**A** | F | WT | 6398.2 |  |
|  |  |  |  |  |  |  | aaGACGGCTACATCCTGGGC**G** | F | Mut | 6414.2 |  |
| 2 |  | | *embB* | 306 | ATG-->CTG | embB306_1_AGC | aaGACGGCTACATCCTGGGC | F | UEP | 6127.0 |  |
|  |  |  |  |  |  |  | aaGACGGCTACATCCTGGGC**A** | F | WT | 6398.2 |  |
|  |  |  |  |  |  |  | aaGACGGCTACATCCTGGGC**C** | F | Mut | 6374.2 |  |
| 2 | Streptomycin (SM) | | *rpsL* | 88 | AAG-->AGG | rpsL88_AG | CTGGTGCGCGGCGGCCGGGTGA | F | UEP | 6849.4 |  |
|  |  |  |  |  |  |  | CTGGTGCGCGGCGGCCGGGTGA**A** | F | WT | 7120.6 |  |
|  |  |  |  |  |  |  | CTGGTGCGCGGCGGCCGGGTGA**G** | F | Mut | 7136.6 |  |
| 2 | Rifampiin  (RIF) | | *rpoB* | 513 | CAA-->AAA | rpoB513_1_CA | GGAGTTCTTCGGCACCAGCCAGCTGAGC | F | UEP | 8590.6 |  |
|  |  |  |  |  |  |  | GGAGTTCTTCGGCACCAGCCAGCTGAGC**C** | F | WT | 8837.8 |  |
|  |  |  |  |  |  |  | GGAGTTCTTCGGCACCAGCCAGCTGAGC**A** | F | Mut | 8861.8 |  |
| 2 |  | | *rpoB* | 513 | CAA-->CCA | rpoB513_2_AC | AGTTCTTCGGCACCAGCCAGCTGAGCC | F | UEP | 8221.3 |  |
|  |  |  |  |  |  |  | AGTTCTTCGGCACCAGCCAGCTGAGCC**A** | F | WT | 8492.5 |  |
|  |  |  |  |  |  |  | AGTTCTTCGGCACCAGCCAGCTGAGCC**C** | F | Mut | 8468.5 |  |
| 2 |  | | *rpoB* | 513 | CAA-->CAG | rpoB513_3_AG | TTCTTCGGCACCAGCCAGCTGAGCCC | F | UEP | 7868.1 |  |
|  |  |  |  |  |  |  | TTCTTCGGCACCAGCCAGCTGAGCCC**A** | F | WT | 8139.3 |  |
|  |  |  |  |  |  |  | TTCTTCGGCACCAGCCAGCTGAGCCC**G** | F | Mut | 8155.3 |  |
| 2 |  | | *rpoB* | 526 | CAC-->TAC | rpoB526_1_CTGA | GAACAACCCGCTGTCGGGGTTGACC | F | UEP | 7668.0 |  |
|  |  |  |  |  |  |  | GAACAACCCGCTGTCGGGGTTGACC**C** | F | WT | 7915.2 |  |
|  |  |  |  |  |  |  | GAACAACCCGCTGTCGGGGTTGACC**T** | F | Mut | 7995.1 |  |
| 2 |  | | *rpoB* | 526 | CAC-->GAC | rpoB526_1_CTGA | GAACAACCCGCTGTCGGGGTTGACC | F | UEP | 7668.0 |  |
|  |  |  |  |  |  |  | GAACAACCCGCTGTCGGGGTTGACC**C** | F | WT | 7915.2 |  |
|  |  |  |  |  |  |  | GAACAACCCGCTGTCGGGGTTGACC**G** | F | Mut | 7955.2 |  |
| 2 |  | | *rpoB* | 526 | CAC-->AAC | rpoB526_1_CTGA | GAACAACCCGCTGTCGGGGTTGACC | F | UEP | 7668.0 |  |
|  |  |  |  |  |  |  | GAACAACCCGCTGTCGGGGTTGACC**C** | F | WT | 7915.2 |  |
|  |  |  |  |  |  |  | GAACAACCCGCTGTCGGGGTTGACC**A** | F | Mut | 7939.2 |  |
| 2 |  | | *rpoB* | 526 | CAC-->CTC | rpoB526_2C_ATG | ACAACCCGCTGTCGGGGTTGACCC | F | UEP | 7314.7 |  |
|  |  |  |  |  |  |  | ACAACCCGCTGTCGGGGTTGACCC**A** | F | WT | 7586.0 |  |
|  |  |  |  |  |  |  | ACAACCCGCTGTCGGGGTTGACCC**T** | F | Mut | 7641.8 |  |
| 2 |  | | *rpoB* | 526 | CAC-->CGC | rpoB526_2C_ATG | ACAACCCGCTGTCGGGGTTGACCC | F | UEP | 7314.7 |  |
|  |  |  |  |  |  |  | ACAACCCGCTGTCGGGGTTGACCC**A** | F | WT | 7586.0 |  |
|  |  |  |  |  |  |  | ACAACCCGCTGTCGGGGTTGACCC**G** | F | Mut | 7602.0 |  |
| 2 |  | | *rpoB* | 526 | CAC-->TGC | rpoB526_2T_AG | CAACCCGCTGTCGGGGTTGACCT | F | UEP | 7016.5 |  |
|  |  |  |  |  |  |  | CAACCCGCTGTCGGGGTTGACCT**G** | F | Mut | 7303.8 |  |
| 2 |  | | *rpoB* | 526 | CAC-->GGC | rpoB526_2G_AG | GCTGTCGGGGTTGACCG | F | UEP | 5258.4 |  |
|  |  |  |  |  |  |  | GCTGTCGGGGTTGACCG**G** | F | Mut | 5545.6 |  |
| 2 |  | | *rpoB* | 526 | CAC-->ACC | rpoB526_2A_AG | ACCCGCTGTCGGGGTTGACCA | F | UEP | 6423.2 |  |
|  |  |  |  |  |  |  | ACCCGCTGTCGGGGTTGACCA**C** | F | Mut | 6670.4 |  |
| 2 |  | | *rpoB* | 522 | TCG-->TTG | rpoB522_2_CTG | GACCAGAACAACCCGCTGT | F | UEP | 5766.8 |  |
|  |  |  |  |  |  |  | GACCAGAACAACCCGCTGT**C** | F | WT | 6014.0 |  |
|  |  |  |  |  |  |  | GACCAGAACAACCCGCTGT**T** | F | Mut | 6093.9 |  |
| 2 |  | | *rpoB* | 522 | TCG-->TGG | rpoB522_2_CTG | GACCAGAACAACCCGCTGT | F | UEP | 5766.8 |  |
|  |  |  |  |  |  |  | GACCAGAACAACCCGCTGT**C** | F | WT | 6014.0 |  |
|  |  |  |  |  |  |  | GACCAGAACAACCCGCTGT**G** | F | Mut | 6054.0 |  |
| 2 |  | | *rpoB* | 522 | TCG-->TTC | rpoB522_3_GC | CCAGAACAACCCGCTGTT | F | UEP | 5428.5 |  |
|  |  |  |  |  |  |  | CCAGAACAACCCGCTGTT**C** | F | Mut | 5675.7 |  |
| 2 | Fluoroquinolone (OFX) | | *gyrA* | 90 | GCG-->GTG | gyrA90_CT | CCACCCGCACGGCGACG | F | UEP | 5126.3 |  |
|  |  |  |  |  |  |  | CCACCCGCACGGCGACG**C** | F | WT | 5373.5 |  |
|  |  |  |  |  |  |  | CCACCCGCACGGCGACG**T** | F | Mut | 5453.4 |  |
| 2 |  | | *gyrB* | 180 | AAC-->GAC | gyrB538_AG | ATGATCGCCTGAACTTCGGTGT | R | UEP | 6741.4 |  |
|  |  |  |  |  |  |  | ATGATCGCCTGAACTTCGGTGT**T** | R | WT | 7068.5 |  |
|  |  |  |  |  |  |  | ATGATCGCCTGAACTTCGGTGT**C** | R | Mut | 6988.6 |  |
| 3 | Rifampin  (RIF) | | *rpoB* | 516 | GAC-->TAC | rpoB516_1A_GT | ACAGCGGGTTGTTCTGGT | R | UEP | 5561.6 |  |
|  |  |  |  |  |  |  | ACAGCGGGTTGTTCTGGT**C** | R | WT | 5808.8 |  |
|  |  |  |  |  |  |  | ACAGCGGGTTGTTCTGGT**A** | R | Mut | 5832.8 |  |
| 3 |  | | *rpoB* | 516 | GAC-->TTC | rpoB516_1T_GT | CAGCGGGTTGTTCTGGA | R | UEP | 5257.4 |  |
|  |  |  |  |  |  |  | CAGCGGGTTGTTCTGGA**A** | R | Mut | 5528.6 |  |
| 3 |  | | *rpoB* | 516 | GAC-->GTC | rpoB516_2AGTC | CGACAGCGGGTTGTTCTGG | R | UEP | 5875.8 |  |
|  |  |  |  |  |  |  | CGACAGCGGGTTGTTCTGG**T** | R | WT | 6202.9 |  |
|  |  |  |  |  |  |  | CGACAGCGGGTTGTTCTGG**A** | R | Mut | 6147.0 |  |
| 3 |  | | *rpoB* | 516 | GAC-->GGC | rpoB516_2AGTC | CGACAGCGGGTTGTTCTGG | R | UEP | 5875.8 |  |
|  |  |  |  |  |  |  | CGACAGCGGGTTGTTCTGG**T** | R | WT | 6202.9 |  |
|  |  |  |  |  |  |  | CGACAGCGGGTTGTTCTGG**C** | R | Mut | 6123.0 |  |
| 3 |  | | *rpoB* | 516 | GAC-->GCC | rpoB516_2AGTC | CGACAGCGGGTTGTTCTGG | R | UEP | 5875.8 |  |
|  |  |  |  |  |  |  | CGACAGCGGGTTGTTCTGG**T** | R | WT | 6202.9 |  |
|  |  |  |  |  |  |  | CGACAGCGGGTTGTTCTGG**G** | R | Mut | 6163.0 |  |
| 3 |  | | *rpoB* | 516 | GAC-->GAG | rpoB516_3_CG | GTCAACCCCGACAGCGGGTTGTTCTG | R | UEP | 7963.2 |  |
|  |  |  |  |  |  |  | GTCAACCCCGACAGCGGGTTGTTCTG**G** | R | WT | 8250.4 |  |
|  |  |  |  |  |  |  | GTCAACCCCGACAGCGGGTTGTTCTG**C** | R | Mut | 8210.3 |  |
| 3 |  | | *rpoB* | 531 | TCG-->CCG | rpoB531_1C_TC | CAGACCGCCGGGCCCCAGCGCCG | R | UEP | 6981.5 |  |
|  |  |  |  |  |  |  | CAGACCGCCGGGCCCCAGCGCCG**A** | R | WT | 7252.7 |  |
|  |  |  |  |  |  |  | CAGACCGCCGGGCCCCAGCGCCG**G** | R | Mut | 7268.7 |  |
| 3 |  | | *rpoB* | 531 | TCG-->GTG | rpoB531_1T_TG | GACCGCCGGGCCCCAGCGCCA | R | UEP | 6363.1 |  |
|  |  |  |  |  |  |  | GACCGCCGGGCCCCAGCGCCA**C** | R | Mut | 6610.3 |  |
| 3 |  | | *rpoB* | 531 | TCG-->GGG | rpoB531_1G_TG | AGACCGCCGGGCCCCAGCGCCC | R | UEP | 6652.3 |  |
|  |  |  |  |  |  |  | AGACCGCCGGGCCCCAGCGCCC**C** | R | Mut | 6899.5 |  |
| 3 |  | | *rpoB* | 531 | TCG-->TTG | rpoB531_2_CTGA | TGACAGACCGCCGGGCCCCAGCGCC | R | UEP | 7598.9 |  |
|  |  |  |  |  |  |  | TGACAGACCGCCGGGCCCCAGCGCC**G** | R | WT | 7886.1 |  |
|  |  |  |  |  |  |  | TGACAGACCGCCGGGCCCCAGCGCC**A** | R | Mut | 7870.1 |  |
| 3 |  | | *rpoB* | 531 | TCG-->TGG | rpoB531_2_CTGA | TGACAGACCGCCGGGCCCCAGCGCC | R | UEP | 7598.9 |  |
|  |  |  |  |  |  |  | TGACAGACCGCCGGGCCCCAGCGCC**G** | R | WT | 7886.1 |  |
|  |  |  |  |  |  |  | TGACAGACCGCCGGGCCCCAGCGCC**C** | R | Mut | 7846.1 |  |
| 3 |  | | *rpoB* | 531 | TCG-->TAG | rpoB531_2_CTGA | TGACAGACCGCCGGGCCCCAGCGCC | R | UEP | 7598.9 |  |
|  |  |  |  |  |  |  | TGACAGACCGCCGGGCCCCAGCGCC**G** | R | WT | 7886.1 |  |
|  |  |  |  |  |  |  | TGACAGACCGCCGGGCCCCAGCGCC**T** | R | Mut | 7926.0 |  |
| 3 |  | | *rpoB* | 533 | CTG-->CCG | rpoB533_2_TC | CGCTCACGTGACAGACCGCCGGGCCCC | R | UEP | 8192.3 |  |
|  |  |  |  |  |  |  | CGCTCACGTGACAGACCGCCGGGCCCC**A** | R | WT | 8463.5 |  |
|  |  |  |  |  |  |  | CGCTCACGTGACAGACCGCCGGGCCCC**G** | R | Mut | 8479.5 |  |
| 3 | Isoniazid (INH) /Ethionamide (ETH) | | *inhA* | - | C-->T (-15) | inhA-15_CT | TATGGGCCACTGACA | F | UEP | 4577.0 |  |
|  |  |  |  |  |  |  | TATGGGCCACTGACA**C** | F | WT | 4824.2 |  |
|  |  |  |  |  |  |  | TATGGGCCACTGACA**T** | F | Mut | 4904.1 |  |
| 3 | Ethambutol (EMB) | | *embB* | 406 | GGC--->GAC | embB406_GA | CCGAGCGCGATGATG | R | UEP | 4618.0 |  |
|  |  |  |  |  |  |  | CCGAGCGCGATGATG**C** | R | WT | 4865.2 |  |
|  |  |  |  |  |  |  | CCGAGCGCGATGATG**T** | R | Mut | 4945.1 |  |
| 3 | Streptomycin (SM) | | *rpsL* | 43 | AAG-->AGG | rpsL43_AG | TACACCACCACTCCGA | F | UEP | 4755.1 |  |
|  |  |  |  |  |  |  | TACACCACCACTCCGA**A** | F | WT | 5026.3 |  |
|  |  |  |  |  |  |  | TACACCACCACTCCGA**G** | F | Mut | 5042.3 |  |
| 3 | Isoniazid  (INH) | | *katG* | 315 | AGC-->ACC | katG315_2_GCA | TAAGGACGCGATCACCA | F | UEP | 5188.4 |  |
|  |  |  |  |  |  |  | TAAGGACGCGATCACCA**G** | F | WT | 5475.6 |  |
|  |  |  |  |  |  |  | TAAGGACGCGATCACCA**C** | F | Mut | 5435.6 |  |
| 3 |  | | *katG* | 315 | AGC-->AAC | katG315_2_GCA | TAAGGACGCGATCACCA | F | UEP | 5188.4 |  |
|  |  |  |  |  |  |  | TAAGGACGCGATCACCA**G** | F | WT | 5475.6 |  |
|  |  |  |  |  |  |  | TAAGGACGCGATCACCA**A** | F | Mut | 5459.6 |  |
| 3 |  | | *katG* | 316 | GGC-->AGC | katG316_GA | TCCATACGACCTCGATGC | R | UEP | 5419.5 |  |
|  |  |  |  |  |  |  | TCCATACGACCTCGATGC**C** | R | WT | 5666.7 |  |
|  |  |  |  |  |  |  | TCCATACGACCTCGATGC**T** | R | Mut | 5746.6 |  |
| 3 | Ethambutol (EMB) | | *embB* | 306 | ATG-->ATC | embB306_3_GCA | ACGGCTACATCCTGGGCAT | F | UEP | 5788.8 |  |
|  |  |  |  |  |  |  | ACGGCTACATCCTGGGCAT**G** | F | WT | 6076.0 |  |
|  |  |  |  |  |  |  | ACGGCTACATCCTGGGCAT**C** | F | Mut | 6036.0 |  |
| 3 |  | | *embB* | 306 | ATG-->ATA | embB306_3_GCA | ACGGCTACATCCTGGGCAT | F | UEP | 5788.8 |  |
|  |  |  |  |  |  |  | ACGGCTACATCCTGGGCAT**G** | F | WT | 6076.0 |  |
|  |  |  |  |  |  |  | ACGGCTACATCCTGGGCAT**A** | F | Mut | 6060.0 |  |
| 3 |  | | *embB* | 497 | CAG-->CGG | embB497_AG | CCAACACCGTTGACAGGGTC | R | UEP | 6087.0 |  |
|  |  |  |  |  |  |  | CCAACACCGTTGACAGGGTC**T** | R | WT | 6414.1 |  |
|  |  |  |  |  |  |  | CCAACACCGTTGACAGGGTC**C** | R | Mut | 6334.2 |  |
| 3 | Fluoroquinolone (OFX) | | *gyrA* | 94 | GAC-->GTC | gyrA94_AG | GGGCCATGCGCACCAGGGTG | R | UEP | 6184.0 |  |
|  |  |  |  |  |  |  | GGGCCATGCGCACCAGGGTG**T** | R | WT | 6511.1 |  |
|  |  |  |  |  |  |  | GGGCCATGCGCACCAGGGTG**A** | R | Mut | 6455.2 |  |

UEP, Unextended probe; WT, Wild-type; Mut, Mutation; F, Forward direction; R, Reverse direction

*, Nucleotides in lower case represent the mismatch to target sequence for the purpose of mass adjustment. The boldface letter in the sequence represent the incorporated nucleotide after single nucleotide extension.

| **Supplemental Table 3.** List of bacteria strains used in MALDI-TOF MS test. | |
| --- | --- |
| **Mycobacterium *tuberculosis* (MTB)** |  |
|  | Mycobacterium *tuberculosis* H37Ra |
| **Non-Tuberculosis Mycobacteria (NTM)** |  |
|  | Mycobacterium *africanum* |
|  | Mycobacterium *bovis* |
|  | Mycobacterium *abscenssus* |
|  | Mycobacterium *fortuitum* |
|  | Mycobacterium *kansasii* |
|  | Mycobacterium *bovis* BCG |
|  | Mycobacterium *gordonae* |
| **Non-Mycobacterium bacteria** |  |
|  | Shigella *sonnei* |
|  | Enterobacter *aerogenes* |
|  | Providencia *alcalifaciens* |
|  | Plesiomonas *shigelloides* |
|  | Vibrio *cholerae* |

**Supplementary Figure 1**


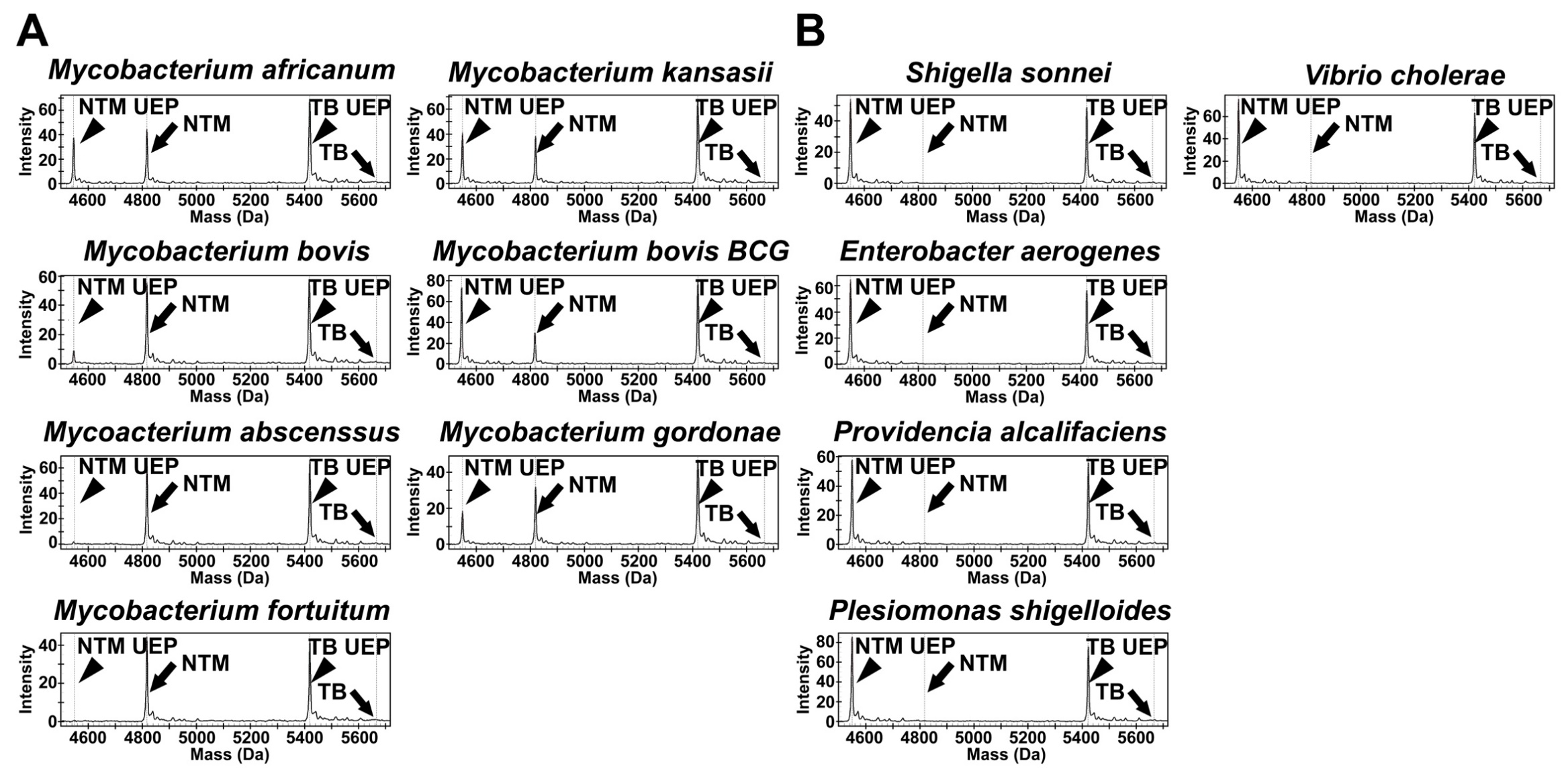


**Reference**

1 Thomas, M. *M. et a*l. Rapid diagnosis of Mycobacterium tuberculosis meningitis by enumeration of cerebrospinal fluid antigen-specific T-cells*. Int J Tuberc Lung D*i**s** 12, 651-657 (2008).
